# Neoplastic human embryonic stem cells as a model of radiation resistance of human cancer stem cells

**DOI:** 10.18632/oncotarget.4165

**Published:** 2015-06-13

**Authors:** Steve Dingwall, Jung Bok Lee, Borhane Guezguez, Aline Fiebig, Jamie McNicol, Douglas Boreham, Tony J. Collins, Mick Bhatia

**Affiliations:** ^1^ McMaster Stem Cell and Cancer Research Institute, Michael G. DeGroote School of Medicine, Faculty of Health Sciences, McMaster University, Hamilton, Canada; ^2^ Department of Biochemistry and Biomedical Sciences, Faculty of Health Sciences, McMaster University, Hamilton, Canada; ^3^ Department of Medical Physics, Faculty of Sciences, McMaster University, Hamilton, Canada; ^4^ David Braley Human Stem Cell Screening Facility, McMaster Stem Cell and Cancer Research Institute, Michael G. DeGroote School of Medicine, Faculty of Health Sciences, McMaster University, Hamilton, Canada

**Keywords:** radiation resistance, cancer stem cells, human stem cells

## Abstract

Studies have implicated that a small sub-population of cells within a tumour, termed cancer stem cells (CSCs), have an enhanced capacity for tumour formation in multiple cancers and may be responsible for recurrence of the disease after treatment, including radiation. Although comparisons have been made between CSCs and bulk-tumour, the more important comparison with respect to therapy is between tumour-sustaining CSC versus normal stem cells that maintain the healthy tissue. However, the absence of normal known counterparts for many CSCs has made it difficult to compare the radiation responses of CSCs with the normal stem cells required for post-radiotherapy tissue regeneration and the maintenance of tissue homeostasis. Here we demonstrate that transformed human embryonic stem cells (t-hESCs), showing features of neoplastic progression produce tumours resistant to radiation relative to their normal counterpart upon injection into immune compromised mice. We reveal that t-hESCs have a reduced capacity for radiation induced cell death via apoptosis and exhibit altered cell cycle arrest relative to hESCs *in vitro*. t-hESCs have an increased expression of BclXL in comparison to their normal counterparts and re-sensitization of t-hESCs to radiation upon addition of BH3-only mimetic ABT737, suggesting that overexpression of BclXL underpins t-hESC radiation insensitivity. Using this novel discovery platform to investigate radiation resistance in human CSCs, our study indicates that chemotherapy targeting Bcl2-family members may prove to be an adjuvant to radiotherapy capable of targeting CSCs.

## INTRODUCTION

Radiation therapy is one of the most important and effective modalities for the treatment of cancer patients with solid tumours and is utilized in approximately 40–50% of all cancer therapies [1]. Technological advances in medical imaging [2] and intensity modulated radiation therapy [3] have greatly improved the capacity to target tumours while limiting normal tissue toxicity, however many patients still suffer from locally recurrent disease after therapy. Such recurrence can occur as late as ten or more years post treatment for some cancers, including breast [4] and prostate [5]. Recent studies have proposed that a subset of cells within several forms of solid tumours, termed cancer stem cells (CSCs), are resistant to radiation relative to the other cells composing a tumour [6–12]. Accordingly, these cells represent an important target for radiotherapy, as failure to eliminate these cells could result in the eventual recurrence of the cancer. However, the genetic and phenotypic heterogeneity of malignant CSCs, as well as the difficulty associated with culturing these cells *in vitro*, limits the capacity to study the response of CSCs to ionizing radiation.

While targeting the CSC population, normal stem cells need to be spared from damage and cell death. Loss of, or damage to, the normal stem cell population can result in an inability to effectively repair tissue post radiotherapy, or could potentially lead to the induction of a secondary cancer. It is therefore necessary to study a normal stem cell counterpart, while devising methods for the successful elimination of cancer stem cells with ionizing radiation. Difficulties in identifying normal counterparts for CSCs, as well as in maintaining both cell types *in vitro*, have limited progress in studying the responses of normal and cancerous stem cells side by side. As a result, a human embryonic stem cell (hESC) line with features of neoplastic progression (herein referred to as t-hESCs) has been characterized [13]. These t-hESCs have been shown to produce neural tumours, which model early human paediatric brain tumours [14]. Importantly these cells have been shown to be effective for identifying biological phenomena unique to human CSCs or cancer stem-like cells [15], when used in conjunction with a normal hESC counterpart, and for identifying therapeutics specific to these CSC populations [16]. Clinically, CSC are considered to exhibit insensitivity to radiotherapy compared to their normal tissue counterpart.

Previously, we have demonstrated that the multi-lineage outgrowths derived from t-hESCs have hallmarks of neoplastic teratocarcinomas with continued present of stem cells [17] or stem-like cells [15] and restricted differentiation-potential in comparison with teratomas derived from normal hESCs [14]. Here, we find t-hESC derived teratocarcinomas are relatively resistant to radiation. This is correlated with the presence of cells staining positive for Oct-4, in these tumours, as well as with decreased levels of apoptosis, and alterations in cell cycle arrest in purified populations of the tumour initiating cells *in vitro*. This model provides a unique system for direct comparison of the radiation response of normal and transformed human stem cells.

## RESULTS

### t-hESCs generate radiation resistant teratocarcinoma upon injection into immune compromised mice

**Figure 1:**
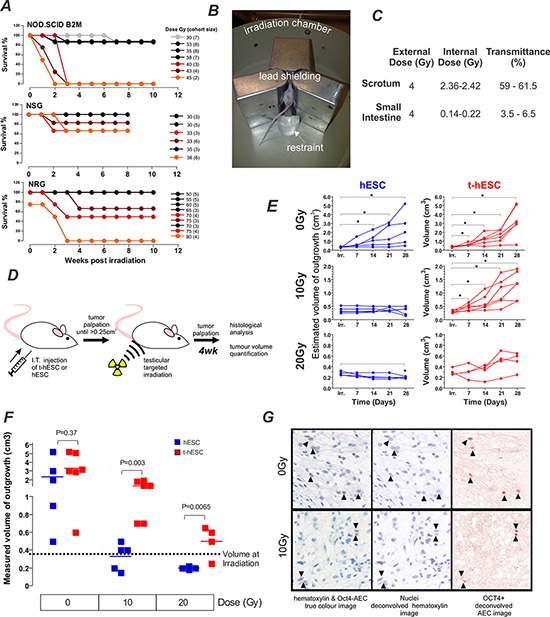
t-hESC derived tumours are radiation resistant compared with their normal counterpart **A.** Comparison of mouse survival for three stains of immunocompromised mice NOD/SCID, NOD/SCID Il2γ−/− (NSG) and NOD Rag1−/− Il2rγ−/− (NRG), used to quantify xenotransplantation post whole body irradiation illustrating relative tolerance to irradiation. Each line represents a separate cohort of mice. Legend describes radiation dose and the number of mice per cohort in parentheses. **B.** Overview of shielding apparatus for targeted irradiation highlighting irradiation chamber, lead shielding and restraint. **C.** Internal dose mice received while in shielding-apparatus measured using thermos-luminescence dosimeter (TLD) chips demonstrating effective shielding of body cavity by shielding apparatus (*n* = 3). **D.** Schematic of experimental design to initiate, radiate and quantify testicular tumours. **E.** Outgrowth (teratocarcinoma from t-hESC or teratoma from hESC) volumes estimated via weekly palpation show no increase in those derived from hESC following either 10 or 20Gy doses gamma-radiation (each series represents 1 mouse). **F.** Final outgrowth (teratocarcinoma from t-hESC or teratoma from hESC) volume was quantified by displacement 4 weeks post irradiation show significantly larger tumours derived from t-hESC compared to hESC. **G.** Immuno-staining post-harvest identified OCT4+ cells in both non-irradiated and 10Gy irradiated teratocarcinoma derived from t-hESCs. Colour deconvolution of the images allowed better visualization of OCT4-positive cells.

Multi-lineage outgrowths form from human pluripotent stem cells when transplanted to immune compromised strains of mice (Supplementary Figure S1). Teratocarcinomas formed from neoplastic hESC are comprised of cells with more primitive lineage than teratomas formed from normal hESC and maintain the ability to form outgrowths in secondary transplants [16]. Our aim was to use this broad-spectrum tumour model to compare the relative radiation-sensitivity *in vivo* of hESC and t-hESC as a surrogate for normal stem cells and radiation-resistance CSCs respectively. The SCID mutation that underlies the immune deficiency in several mice strains has previously been shown to cause general defects in DNA repair [18]. We hypothesized that mice containing the SCID mutation might be hypersensitive to radiation, thus limiting their utility as recipients for the assessment of the radiation sensitivity of transplanted cells. To identify the optimal strain of mouse for our studies, i.e. with the lowest radiation-sensitivity, we analysed previous radiation exposure results from independent and unrelated studies on the effects of irradiation on three strains of immunocompromised mice–two with the SCID mutation (NOD.SCID and NSG) and one without (NRG). Strains harbouring the SCID mutation exhibited increased mortality at doses less than 50 Gy (Figure 1A). In comparison NRG mice, with immune deficiency mediated through Rag1 deletion rather SCID mutation, did not show increased mortality at doses of 65Gy or less. To further minimise the effects of radiation on the recipient and maximize the dose that could be delivered to the tumours, lead shielding was constructed to localize the irradiation (Figure 1B). To ensure the shielding was effective in reducing non-targeted radiation, and quantify the internal dose received, to the site of injection, thermo-luminescence dosimeter (TLD) chips were surgically implanted into the scrotum and small intestine of mice. The shielding reduced the radiation reaching the small intestine to 3.5–6.5% of the external dose while only partially reducing the radiation reaching the testes to 59–61.5% (Figure 1C).

Using this experimental configuration, the effect of irradiation on growth of teratocarcinomas derived from t-hESC and teratomas from hESC was quantified (Figure 1D). After intra-testicular injection with either hESC or t-hESC, mice were palpated weekly and irradiated when outgrowth volume reached 0.25–0.45 cm^3^. Estimation of volume by palpation showed that hESC derived teratomas ceased to continue growing after exposure to 10Gy and 20Gy irradiation and teratomas irradiated with 20Gy had significantly shrunk (paired- *t*-test *p* = 0.002) from their initial size. Teratocarcinomas derived from t-hESC however, did not cease to grow after exposure to 10Gy irradiation (Figure 1E) and with 20Gy irradiation did not show the significant reduction in size seen with hESC-derived teratomas. Four weeks later outgrowths were harvested and more precisely quantified by displacement. There was no significant difference in volume between non-irradiated outgrowths derived from t-hESC and hESCs. However, t-hESC derived teratocarcinomas were significantly larger than hESC derived teratomas following both 10Gy (*p* < 0.01) and 20Gy (*p* < 0.01) doses (Figure 1F). These results demonstrate that teratocarcinomas derived from t-hESCs relatively resistant to radiation when compared to their normal hESC counterpart.

A key characteristic of cancer stem cells is their ability maintain self-renewal capacity to initiate disease upon serial transplant [19]. To determine whether the self-renewing fraction of the teratocarcinomas was affected by the radiation they were analysed via immunohistochemistry for the presence of the pluripotency marker OCT4. Detection of OCT4 in teratocarcinomas derived from t-hESC has previously been correlated with their unique ability to form secondary tumours [16]. The presence of OCT4 positive cells in teratocarcinomas initiated by t-hESCs is indicative of the maintenance of self-renewal capacity following irradiation (Figure 1G). These results demonstrate a relative insensitivity to radiation in the self-renewing fraction of t-hESC *in vivo*, which is consistent with that seen clinically in primary CSCs.

### The relative radiation-sensitivity of t-hESCs and hESCs observed *in vivo* is recapitulated *in vitro*

**Figure 2:**
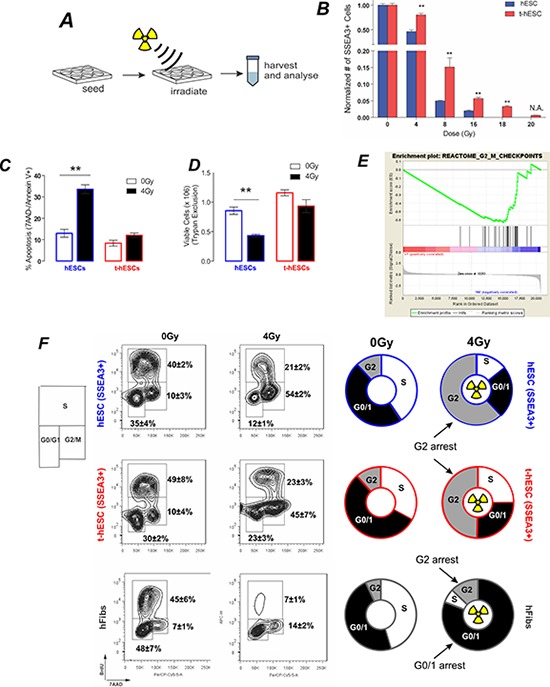
*In vivo* radiation resistance of t-hESC is recapitulated *in vivo* and results in G2 arrest **A.** Schematic of experimental design for characterisation *in vitro* radiation response. **B.** Total SSEA3+ cell count 72 hours post irradiation of hESC and t-hESC cultures over a range of doses reveals a greater decrease in the number of SSEA3+ hESC relative to t-hESCs at all doses of radiation (*n* = 3). **C.** Relative percentages of cells in early apoptosis (7AAD-, Annexin V +) show an increase in the induction of apoptosis in hESC (SSEA3+) cells relative to t-hESC (SSEA3+) cells (*P* < 0.01, *n* = 4) 8 hours after exposure to a 4 Gy dose of γ. **D.** Relative number of viable hESC and t-hESC cells 8 hours after exposure to a 4 Gy dose of γ irradiation measured via trypan blue shows an increase in cell death in hESC cells relative to t-hESC cells (**, *P* < 0.01, *n* = 4). **E.** GSEA of G2-M checkpoint genes show an enrichment in hESC vs t-hESC. **F.** Flow cytometric quantification of cell cycle through 7AAD staining (DNA) and BrdU incorporation 12 h post-irradiation for SSEA3+ h-ESC, SSEA3+ t-hESC and fibroblasts. Both hESC and t-hESC preferentially arrest in G2 while fibroblasts arrest predominately in G1/0 and to a lesser extent in G2 although hESC show a significantly greater frequency of cells in G2 (*p* < 0.01, *n* = 4).

Unlike primary CSC samples, our model system affords the unique capacity for long culture of an enriched human cancer stem cell population facilitating mechanistic studies of CSC self-renewal and differentiation term *in vitro* [16]. To determine whether CSC radiation-resistance was also replicated *in vitro*, we compared the response of the self-renewing fraction of hESC and t-hESC cultures to irradiation (Figure 2A). For *in vitro* cell studies the pluripotency cell-surface marker SSEA3 or transcription factor OCT4 were used to identify the pluripotent cell fraction.

Both hESC and t-hESC showed a reduction in SSEA3+ cell counts 72 hours following all doses of Cs-137 γ radiation tested. hESCs exhibited a greater sensitivity to irradiation though, with significantly greater decrease in the number of SSEA3+ cells relative to t-hESC cultures for all treatment (Figure 2B). To minimize the effects of cellular necrosis on apoptosis induction, a low radiation dose (4 Gy) was used to quantify the apoptotic response. 4 hours following exposure to a 4Gy dose of Cs-137 gamma-radiation resulted in an approximately three-fold increase in the proportion of normal SSEA3+ hESCs undergoing apoptosis (Annexin V+, 7AAD-), whereas a significant increase was not observed in SSEA3+ t-hESCs (Figure 2C). Additionally, the proportion of cells undergoing apoptosis was associated with a decreased number of viable cells in only hESCs and not t-hESCs (Figure 2D). This demonstrates that the relative insensitivity of t-hESCs to radiation observed *in vivo* is recapitulated *in vitro*, thereby providing a unique model system to study radiation resistance.

### t-hESCs exhibit a mechanism of radiation-resistance previously undescribed in CSCs

**Figure 3:**
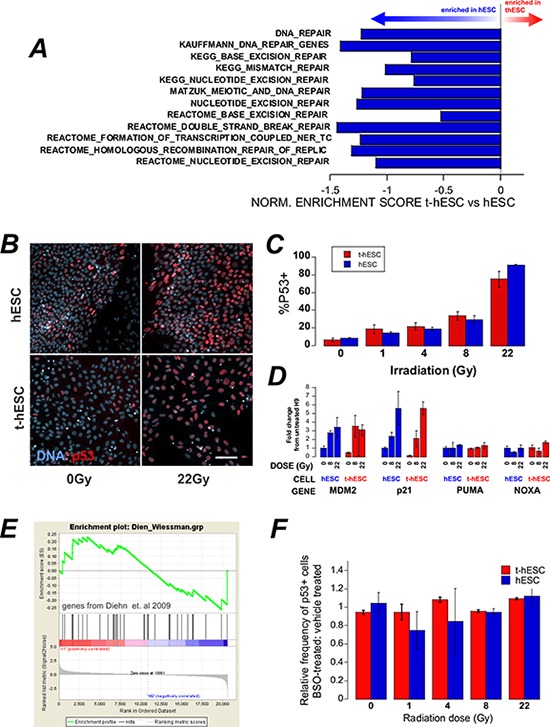
A. GSEA of a number of genesets related to DNA repair show an enrichment in hESC over t-hESC **B.** *In situ* staining of p53 in hESC and t-hESC post-irradiation. **C.** Quantification of the increase in p53 positivity in reponse to increasing doses of radiation showed no significant difference between t-hESC and hESC (means ± sem, *N* = 2, *n* = 3). **D.** qPCR analysis of transcriptional targets of p53 8 hr post irradiation. **E.** GSEA of genes implicated in reactive-oxygen species processing in radio-resistant breast-cancer stem cells over bulk tumour cells shows no enrichment between t-hESC and hESC. **F.** t-hESC were pre-incubated with 1 mM BSO to reduce the ROS-buffering capacity of the cells for 24 hrs prior to irradiation with increasing doses of gamma radiation. The frequency of p53 positive cells was unaffected. Means ± sem, *n* = 3.

DNA damage post irradiation results in cell cycle arrest; attempted DNA repair; followed by a decision between apoptosis or re-entry to the cell cycle. Following exposure to ionizing radiation somatic cells undergo cell cycle arrest at both the G1 and G2 checkpoints [20], however the G1 checkpoint has been shown to be absent in hESCs [21]. Geneset enrichment analysis (GSEA) of global expression data for the two cell lines showed significant (*p* < 0.01) enrichment genes associated with G2/M checkpoint in hESCs compared to t-hESCs suggesting that normal hESCs may have altered progress through the G2/M checkpoint post-irradiation (Figure 2E). To determine whether this equated to a functional defect that may explain the radiation resistance, cell cycle analysis was performed on control and irradiated cells. hESCs, t-hESCs and fibroblasts were incubated for 1 hour with the thymidine analog bromodeoxyuridine (BrdU) and stained for DNA content with 7AAD, for analysis of the cell cycle response in the SSEA3+ population following ionizing radiation (Figure 2F). Consistent with the absence of a G1 checkpoint, both hESC and t-hESC preferentially arrested in G2 when compared with an immortalized somatic fibroblast cell line (AGO1522) 12 hours post irradiation. However, although t-hESCs exhibited G2 arrest post irradiation, they exhibited a lower frequency of cells in G2 and greater in G0/1 (*p* < 0.05, *n* = 4) than hESCs (Figure 2F). Enhanced DNA repair post irradiation has been described in glioma stem cell compared to the rest of the glioma tumour [6] raising the possibility of the reduced G2 arrest in t-hESC is due to expedited repair and exit from arrest. However, GSEA of twelve separate gene-lists associated with DNA repair suggest that t-hESCs are less enriched (normalised enrichment score less than zero) for DNA repair genes compared with normal hESCs (Figure 3A).

The higher percentage of SSEA3+ hESCs in G2/M post irradiation compared with SSEA3+ t-hESCs could alternatively arise due to an increase in the number of hESCs failing to re-enter the cell cycle and progressing towards apoptosis. To better understand the relationship between cell cycle response vs. apoptosis, we examined p53. Previous studies have established the involvement of p53 in the activation of multiple cellular responses to ionizing radiation, including both cell cycle response and apoptosis (reviewed by [22]) we investigated whether t-hESCs exhibited altered nuclear p53 accumulation response to irradiation compared with pluripotent (Oct4+) hESCs. However, *in situ* staining and quantitative imaging demonstrated no difference in the frequency of p53 positive cells in t-hESCs and pluripotent hESCs cells in response to increasing doses of radiation (Figure 3B–3C). Similarly, the transcriptional targets of p53 related to p53-regulation and cell cycle arrest (MDM2 and p21) were equally upregulated in hESCs and t-hESCs eight hours post irradiation (Figure 3D). However, at this time-point other transcriptional targets of p53 related to apoptosis (PUMA, NOXA) were not upregulated in either cell-type.

In a comparison between the stem cell and non-tumour initiating compartment of tumours, radiation-resistance in mammary CSCs was suggested to result from enhanced processing of reactive oxygen species (ROS) by CSCs resulting in decreased cellular damage for a given radiation dose [11]. Geneset enrichment analysis of the key ROS-scavenging genes linked in this study to radiation-resistance showed in no significant enrichment in t-hESCs compared with hESCs (Figure 3E). Furthermore, unlike breast cancer stem cells, pre-incubation of t-hESCs for 24 hour prior to irradiation with 1 mM L-Buthionine Sulfoxide (BSO) to deplete cellular glutathione did not result in sensitization – as indicated by no in increase in p53 positivity at a lower radiation dose (Figure 3F). Together, this data suggests that ROS-scavenging is not the mechanism of radiation-resistance in t-hESCs over hESCs.

**Figure 4:**
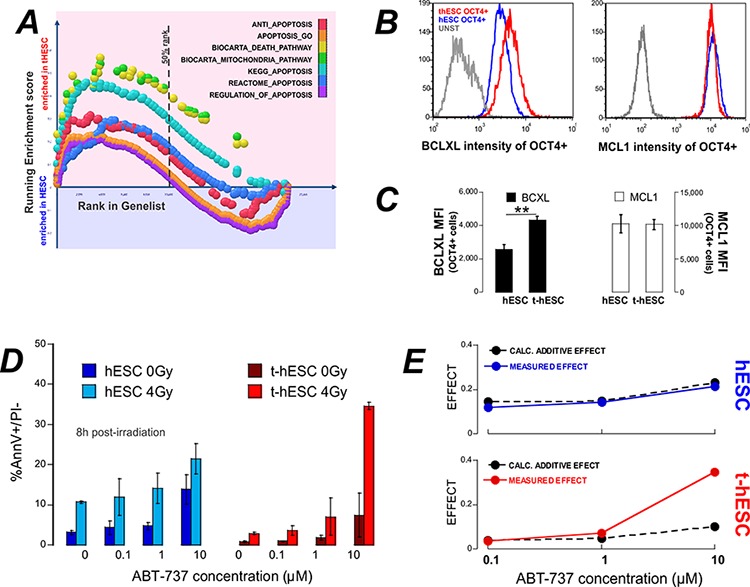
t-hESC are enriched for apoptosis and treatment with the BH3-mimetic ABT737 results in enhanced radio-sensitivity **A.** GSEA of genelists related to apoptosis show enrichment in t-hESCs over hESCs. **B.** and **C.** Quantitative flow cytometry of BclXL and MCL1 show significantly increased expression of BclXL in t-hESC. **D.** hESC and t-hESCs were treated with ABT737 over a range of concentrations and irradiated at threshold dose of radiation (4 Gy). Apoptosis was quantified 8 hrs post irradiation (*n* = 3). **E.** Calculation of the theoretical additive effect of irradiation and measured effect of shows synergy between ABT737 and irradiation in t-hESCs only.

GSEA of global expression profiles of untreated cells demonstrated enrichment in t-hESCs over hESCs in 7 out of 7 apoptosis related genesets (Figure 4A). In particular, BCL2L1 – the gene encoding BclXL – was identified in multiple genesets as a core enrichment gene. Flow cytometry analysis revealed significantly elevated (*p* < 0.01, *n* = 3) BclXL protein levels in t-hESCs compared to hESCs (Figure 4B). As a control, a second anti-apoptosis gene, MCL1, that has been associated with self-renewal of the hematopoietic stem cell compartment [23], was compared between t-hESCs and hESCs. MCL1 was not flagged as core-enrichment gene and immuno-staining revealed no different the two lines (Figure 4C–4D). Since overexpression of anti-apoptosis members of the Bcl2-family has been linked to chemo-resistance in colon cancer stem cells [24] we investigated whether inhibition of BclXL would re-sensitize t-hESCs to radiotherapy. Both hESCs and t-hESCs received a 4 Gy dose of radiation in the presence of increasing concentrations of the BH3-mimetic ABT737 [25]. Eight hours post-irradiation the frequency of apoptotic cells (AnnV+/PI−) was quantified. Both hESC and t-hESC showed a significant dose dependent increase in the frequency of apoptotic cells in response to ABT737 treatments in the absence of irradiation (vehicle vs 0.1 μM ABT737; *p* < 0.05, *n* = 3). The combination of irradiation and ABT737 treatment resulted in a significant (*p* > 0.05, *n* = 3) increase apoptosis in both t-hESC and hESCs over ABT737 treatment alone (Figure 4D). To determine whether the combination of ABT737 and radiation treatment were additive or synergistic, we calculated the fractional-effect of treatment combinations [26]. Synergy between two treatments is clinically desirable and identified as a greater effect than the product of each of the individual treatment alone. For hESCs the calculated additive effect matched the observed effect. For t-hESCs however, there is evidence of synergy between ABT737 and radiation where the observed effect of 10 μM ABT737 treatment and irradiation was over three times (3.4x) that predicted by fractional analysis (Figure 4E).

## DISCUSSION

The primary goal of this work was to evaluate the radiation response of normal vs. human CSCs towards potentially identifying relationships to radiation resistance observed in multiple CSC populations in patients. Our group is the first to utilize a severely immune deficient mouse capable sustaining complex human tumour growth as well as surviving clinically relevant targeted doses of irradiation resulting in a unique mode of quantification of radiation sensitivity of human tissues *in vivo.* This mode of quantification of the *in vivo* radiation sensitivity has great potential utility for studying the radiation response of multiple types of cancer xenografts. Further, we have for the first time directly compared the radiation response of a transformed stem cell to its normal counterpart and found that the normal counterpart is more sensitive to radiation.

Radiation resistant tumours are a serious obstacle for the clinical treatment of multiple types of cancers including breast [27], head and neck [28], and prostate [29]. Enhanced radiation resistance has been observed in multiple CSC populations, relative to non-CSCs residing in the same tumour [6–12]. However, due to difficulty maintaining CSCs and difficulty identifying normal counterparts for analysis *in vitro*, no comparison with a normal stem cell counterpart has been made to date. This comparison is essential for the identification therapeutics that will target CSCs without impacting the associated healthy tissue. Our study demonstrates that normal vs. CSCs capable of giving rise to multiple tissue types *in vivo* have different sensitivities to radiotherapy as demonstrated by xenotransplantation. This was only possible with the use of NRG recipient mice, where the DNA-repair defect that underlies immune-deficiency is localised to the immune-system; allowing local damage of recipient tissues that can repair post radiotherapy and not confound the human normal or CSC progeny. Irradiation of teratoma outgrowths derived from normal hESCs is effective at stopping, and even reversing, growth. However, we have previously demonstrated that the multi-lineage outgrowths derived from t-hESCs have hallmarks of neoplastic teracarcinomas with higher self-renewal that teratomas and poor differentiation [13, 16] and we now find teratocarcinomas from t-hESC are less sensitive to radiotherapy and continued to contain cells that expressed Oct-4, suggesting the maintenance of multi-potent population within the tumour following radiation. This is analogous to the proposed role of CSCs in failed radiotherapy attempts and the subsequent recurrence of tumorigenesis [30, 31]. However, unlike CSC populations exhibiting radiation resistance isolated from primary tumours and cell lines, we were able to culture these cells and compare them directly with a counterpart which is capable of producing heterogeneous tumours that are not resistant to radiation for further testing, thereby capturing human CSC radiation-resistance seen *in vivo* was recapitulated in an *in* vitro system that is amenable to further characterisation.

The ability to easily culture t-hESCs *in vitro* provided a unique opportunity for mechanistic studies of radiation resistance, as well as an opportunity for the screening of potential CSC radiation-sensitizing agents. Using this *in vitro* system we reveal that t-hESCs representing CSCs have aberrant apoptotic and cell cycle responses following irradiation. As previously observed, while ionizing radiation results in cell cycle arrest at both the G1 and G2 phases of the cell cycle in proliferating somatic cells [32], the self-renewing fraction of hESCs and t-hESCs predominantly arrest in G2 [21]. Here we report for the first time differences in cell cycle arrest between SSEA3+ hESCs and t-hESCs and point to a shortened G2 arrest in transformed stem cells. A shortened arrest possibly points to more efficient DNA-damage repair, as suggested for glioma cancer stem cells [6]. Another possible mechanism of radiation-resistance in CSCs is enhanced ROS scavenging, as observed in breast CSCs [33]. However, neither of these mechanisms appear to underlie the radiation resistance of t-hESCs over hESCs and may reflect the difference in comparisons made between CSCs and other tumour cells and comparisons between CSCs and normal stem cells. Instead we observed that radiation resistance of t-hESCs was associated with enhanced expression of BclXL. A role for BclXL has also been implicated in cell cycle modulation [34] and possibly underpins the differences in cell cycle arrest between normal and t-hESCs we have observed post irradiation.

Both h-ESC and t-hESC respond to irradiation with accumulation of p53 and transcriptional activation of cell cycle related genes although some pro-apoptotic transcriptional targets of p53 are not upregulated. Aside from transcriptional activation of pro-apoptotic genes, p53 is also thought to directly activate apoptosis through release of pro-apoptostic proteins BAX and BAK from pre-existing complexes with anti-apoptotic BclXL and Bcl2 [35]. Failure for NOXA and PUMA transcript to increase post p53 accumulation points towards a non-transcriptional role for p53 in hESC and t-hESC apoptosis. Our data is consistent with the balance between pro- and anti-apoptotic proteins being skewed in t-hESC towards survival post-irradiation through overexpression of BclXL. This balance could be restored towards cell-death by the BclXL inhibitor ABT737. A role for BclXL has also been implicated in cell cycle modulation [34] suggesting a possibly role in the differences in cell cycle arrest post-irradiation between normal.

Collectively, our results implicate the need for molecules/techniques that will help to either enhance the ability to eliminate transformed stem cells relative to normal stem cells, or to protect only normal stem cells from damage. The capacity to isolate a purified population of radiation-resistant human transformed stem cells *in vitro*, and directly compare them to a normal counterpart, might provide an ideal platform for the identification of such molecules/techniques.

## MATERIALS AND METHODS

### Animals

Animal experiments were approved by the Animal Care Committee (Animal Research Ethics Board), and Veterinary Services of McMaster University. Approval from our local ethics board was obtained for use of established human embryonic stem cell lines and SCOC.

### Human embryonic stem cell culture

Human Embryonic Stem Cell Culture was performed as previously published (Chadwick et al, 2003; Stewart et al., 2006; Werbowetski-Ogilvie et al., 2009). Briefly, normal (H9; hESC) and transformed (v-H9–1, t-hESC) hESC cell lines were cultured on Matrigel (BD Biosciences) coated plates in Mouse Embryonic Fibroblast Conditioned Media (MEF-CM) supplemented with 8ng/ml of basic fibroblast growth factor (bFGF) (Invitrogen). MEF-CM was produced in house by daily collection of media used to feed irradiated mouse embryonic fibroblasts (MEFs) over a 7 day period. Media used to feed MEFs consisted of 80% knockout Dulbecco modified eagle medium, 20% knockout serum replacement, 1% non-essential amino acids, 1uM L-glutamine (all Invitrogen), 0.1 mM β-mercaptoethanol (Sigma Aldrich), and 4 ng/mL bFGF. After collection MEF-CM was filtered through a 0.22-μM and stored at −30°C. Cells were dissociated for 2–5 min with collagenase IV (Gibco) and passaged at a 1:6 ratio every 4 days (t-hESCs) or at a 1:2 ratio every 7 days (hESCs). Cells were maintained in a 37°C incubator with 5% CO2.

### Gene set enrichment analysis

Total RNA from t-hESC and h-ESC was extracted using total RNA isolation kit (Norgen Biotech) and hybridized to Affymetrix Human Gene 1.0 ST arrays (London Regional Genomics Centre). Raw expression data was (RMA) normalized using GenePattern 2.0 [36](Broad Institute) for Gene Set Enrichment Analysis [37, 38].

### *In vitro* irradiation

7 (hESCs) or 4(t-hESCs) days following cell passaging, media was aspirated until just covering cells. Cells were irradiated at room temperature using a 137Cs (662Kev) γ ray source (McMaster Taylor Source), at a distance of 30cm, resulting in a dose rate of 0.344Gy/min. Following irradiation, fresh media was added to cultures and cells were returned to incubator.

### Cell harvest and SSEA3 staining

hESCs and t-hESCs were treated with collagenase IV and then dissociated in cell dissociation buffer for 10 min at 37°C. Cells were then resuspended in 4mL of PBS/3%FBS and centrifuged at 1500rpm for 5 min. Supernatant was aspirated and cells were again resuspended in of PBS/3%FBS and filtered through a 40 um filter. Cell counts were measured using an automated cell counter (Countess, Invitrogen) and diluted to a concentration of 1 × 106 cells/mL. Cell suspensions were then conjugated with PE-SSEA3(1:100) (BD Bioscience) for 30 min at room temperature, followed by two washes with PBS/3%FBS as above.

### Apoptosis and cell cycle analysis

Cells were harvested 8 hrs post irradiation and stained for SSEA3, as above, then resuspended in 1x Binding Buffer (BD Bioscience). Cell suspensions were then conjugated with FITC-Annexin V (1:20) for 15 min. The DNA stain 7-Amino-actinomycin D (7-AAD) (BD Bioscience) was added (1:100) and cells were incubated at room temperature for 15 min. Analysis was performed using a FACSCalibur (BD Bioscience) and FlowJo software (Tree Star). Day 7 hESCS and day 4 t-hESCs were exposed to 10 μM BrdU for 1 hour, prior to cell harvest. Cells were harvested either 12 or 72 hrs post irradiation and stained for SSEA3, as above. Cells were then washed with 1 ml of Perm/Wash buffer (BD Bioscience) and centrifuged at 1500 rpm for 5 min and then fixed in Cytofix/Cytoperm Plus (BD Bioscience) for 10 min at room temperature. Cells were again washed with Perm/Wash buffer then fixed with Cytofix/Cytoperm buffer (BD Bioscience) for 5 min at room temperature. Perm/Wash buffer was repeated then cell pellets were resuspended in DNAase (300 ug/mL) (BD Bioscience) and incubated at 37°C for 70 min. Cells were then washed with Perm/Wash buffer and conjugated with APC-antiBrdU (1:50) (BD Bioscience), for 20 min at room temperature. Cells were then stained for DNA content with 7AAD (1:10) (BD Bioscience) at room temperature for 10 min prior to analysis. Analysis was performed using a FACSCalibur (BD Bioscience) and FlowJo software (Tree Star).

### Immuno-cytochemistry staining, automated imaging, and analysis

Cells were fixed in 2% paraformaldehyde and stained with 10 μg/mL Hoechst 33342 (Invitrogen) with a Combi Multidrop Dispenser (Thermo). Standard fluorescence immune-cytochemical techniques were used to stain the cells with a monoclonal antibodies for p53 (Cell Signaling Technologies) and for Oct4 (BD), and Alexa-Fluor-488 and Alexa-Fluor-546 secondary antibodies (Invitrogen). Immuno-cytochemical staining was performed by a Janus automated liquid handler (Perkin Elmer). Images were acquired at 20x with an Operetta (Perkin Elmer) using standard filter sets. Image analysis was performed using custom scripts in Acapella software (Perkin Elmer). Nuclear objects were segmented from the Hoechst signal. The fraction of nuclear-localised Alexa-Fluor-488- and Alexa-Fluor-546-positive cells was quantified. Images and well-level data were stored and analysed in a Columbus Database (Perkin Elmer).

### Xenotranplantation, *in vivo* irradiation, and histology

hESCs (200K) and t-hESCs (50K) were treated with collagenase IV for 3–5 min, as described above, and resuspended in 25 ul of PBS/3%FBS. Cell suspensions were injected intratesticularly into NOD.Rag1−/− IL-2rγc−/− (NRG) mice (Jax Laboratories). Injected and control testicles were palpated weekly and measured using callipers, beginning 3 weeks post injection and continuing until tissue collection. Irradiations were performed when the palpated volume of the injected testicle reached a 0.25–0.45 cm3.

*In Vivo* Irradiations were performed using a Cs-137 γ irradiation source (Gammacell 40 Exactor, MDS Nordion) at a dose rate to the skin of 0.97Gy/min. Mice were anesthetised using 2.5% Avertin (Sigma Alrich) (0.018 ml/g body mass) and placed in lead shielding to localize the dose to the testicles. Measurements of direct radiation dose to the inside of the testicles and indirect dose received in the small intestines, while mice were housed in lead shielding, were obtained by surgical implantation of thermo-luminescence dosimeter (TLD) chips (Harshaw TLD-100 LiF Chips). TLD chip analyses were performed by a third party specializing in clinical diagnostic radiation measurements (K&S Associates Inc., Nashville, TN). Mice were euthanized 4 weeks following irradiation. Tumours were extracted and volume was measured by displacement of 10% formalin in a graduated cylinder and with callipers. The weight of all tumours was also measured and recorded. Tumours were fixed in 10% formalin, imbedded in paraffin and sectioned in 5 um intervals. This was followed by depraffinization in xylene and dehydration in a graded series of ethanol concentrations. Samples were stained with H&E or Oct4 (1:200). Histological samples were scanned at 200x and 400x magnification with an automated slide scanner (Scanscope CS, Aprio) and analysed with ImageJ.

### Statistical analysis

All tests were performed using Prism 5 software (GraphPad Software, La Jolla, CA, USA). Descriptive statistics were used to determine significant differences including mean and s.e.m. along with one-way ANOVAs and independent sample two-tailed *t*-tests. *P*-values < 0.05 were considered significant.

## SUPPLEMENTARY FIGURE


