# Supplementary material for: Neoplastic human embryonic stem cells as a model of radiation resistance of human cancer stem cells
Source: Oncotarget. 2015 Jun 13;6(26):22258–69. doi: 10.18632/oncotarget.4165 (PMC4673161; doi:10.18632/oncotarget.4165)
Supplement: Supplementary file 1 [file oncotarget-06-22258-s001.pdf]

## SUPPLEMENTARY FIGURE

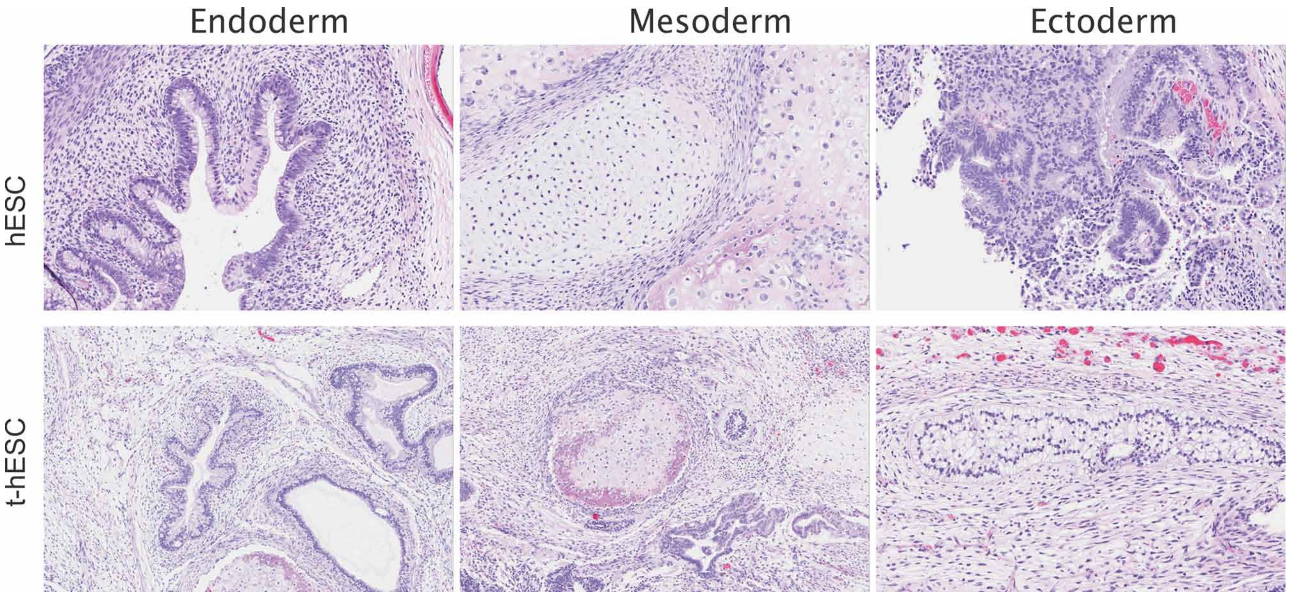


**Supplementary Figure S1: Immuno-histochemical analysis of teratoma or teratocarcinoma derived from xeno-transplanted hESC and t-hESCs respectively illustrate multi-lineage potential with the generation of tissue from three germ layers.**
